# A novel method for maize leaf disease classification using the RGB-D post-segmentation image data

**DOI:** 10.3389/fpls.2023.1268015

**Published:** 2023-09-26

**Authors:** Fei Nan, Yang Song, Xun Yu, Chenwei Nie, Yadong Liu, Yali Bai, Dongxiao Zou, Chao Wang, Dameng Yin, Wude Yang, Xiuliang Jin

**Affiliations:** ^1^ College of Agriculture, Shanxi Agricultural University, Taigu, Shanxi, China; ^2^ Institute of Crop Sciences, Chinese Academy of Agricultural Sciences/Key Laboratory of Crop Physiology and Ecology, Ministry of Agriculture, Beijing, China; ^3^ National Nanfan Research Institute (Sanya), Chinese Academy of Agricultural Sciences, Sanya, China

**Keywords:** leaf spot, disease classification, deep learning, convolutional neural network, depth camera, image processing, smart agriculture, crop breeding

## Abstract

Maize (*Zea mays* L.) is one of the most important crops, influencing food production and even the whole industry. In recent years, global crop production has been facing great challenges from diseases. However, most of the traditional methods make it difficult to efficiently identify disease-related phenotypes in germplasm resources, especially in actual field environments. To overcome this limitation, our study aims to evaluate the potential of the multi-sensor synchronized RGB-D camera with depth information for maize leaf disease classification. We distinguished maize leaves from the background based on the RGB-D depth information to eliminate interference from complex field environments. Four deep learning models (i.e., Resnet50, MobilenetV2, Vgg16, and Efficientnet-B3) were used to classify three main types of maize diseases, i.e., the curvularia leaf spot [*Curvularia lunata* (Wakker) Boedijn], the small spot [*Bipolaris maydis* (Nishik.) Shoemaker], and the mixed spot diseases. We finally compared the pre-segmentation and post-segmentation results to test the robustness of the above models. Our main findings are: 1) The maize disease classification models based on the pre-segmentation image data performed slightly better than the ones based on the post-segmentation image data. 2) The pre-segmentation models overestimated the accuracy of disease classification due to the complexity of the background, but post-segmentation models focusing on leaf disease features provided more practical results with shorter prediction times. 3) Among the post-segmentation models, the Resnet50 and MobilenetV2 models showed similar accuracy and were better than the Vgg16 and Efficientnet-B3 models, and the MobilenetV2 model performed better than the other three models in terms of the size and the single image prediction time. Overall, this study provides a novel method for maize leaf disease classification using the post-segmentation image data from a multi-sensor synchronized RGB-D camera and offers the possibility of developing relevant portable devices.

## 1 Introduction

As one of the major crops in the world, maize (*Zea mays* L.) is not only an essential source of feed for livestock, aquaculture, and the fisheries industry, but also an indispensable raw material in food, healthcare, light industry, and chemical industries (Olawuyi et al., 2014). In recent years, maize diseases have become increasingly severe, seriously affecting the yield and quality of maize. Therefore, breeding new varieties with strong disease resistance, high yield, and excellent quality has become one of the vital approaches to improve maize yield and quality (Song et al., 2021), which requires maize disease classification thus to accurately identify germplasm for breeding. However, the current judgment for maize leaf diseases in germplasm identification and breeding primarily relies on the experience of experts. This requires high professional knowledge, but efficiency is relatively low, which is the reason maize germplasm identification at large-scale and new variety breeding has become a bottleneck (Fang et al., 2022). The development of sensors and data analysis methods. However, the existing crop disease recognition still has low accuracy and poor targeting. In addition, the existing crop disease recognition has a variety of problems, such as low accuracy, poor targeting, etc., which cannot meet the actual needs of corn growers. Deep learning, as an emerging technology in the field of machine learning, has a wide range of applications in the field of image recognition. Consequently, there is an urgent need to develop a new method to identify disease information quickly and accurately in complex field environments.

In recent years, object recognition and classification research based on the convolutional neural network (CNN) has gradually become a hotspot (Cap et al., 2022), which has been widely applied in fields like animal husbandry, agriculture, and land use classification (Priyadharshini et al., 2019; Hitelman et al., 2022; Campos-Taberner et al., 2023). For example, Zeng et al. (2022) proposed improving the SKPSNet-50 models to classify eight kinds of maize leaf diseases. Agarwal et al. (2020) developed an efficient CNN model for tomato disease identification and achieved 93.5% accuracy. Azgomi et al. (2023) designed a multi-layer neural network for classifying four diseases with low computation cost at only. However, the classification performance was not competitive with an accuracy of 73.70%. Kumar et al. (2022) proposed an automatic system for tomato leaf disease recognition based on the deep convolutional neural network (DCNN). Ma et al. (2023) proposed an improved YOLOv5n model to identify common maize leaf spots, gray spots, and rust diseases in mobile applications. The average recognition accuracy of the model reached 95.2%, which was 2.8% higher than the original model. In addition, the memory size was reduced to 5.1 MB compared to 92.9 MB of YOLOv5l, which was 94.5% smaller, meeting the light weighting requirement. Sun et al. (2021) proposed an improved InceptionV3 model based on deep data with a maturity class classification accuracy of up to 98%. The depth data can reflect the characteristics of flower buds well, which helps to classify the maturity grade. Fei et al. (2023) proposed an improved ShuffleNet V2 for fresh cut flowers classification, which can achieve a classification accuracy of 99.915% in the RGB-D flowers dataset, with an overall predicted classification speed of 0.020 seconds per flower. Compared with the fresh-cut flower classifiers currently available in the market, this method has a great advantage in speed.

Current research trends in classification networks can be categorized into two main types: 1) Pursuing higher performance, which often results in an increase in the number of model parameters and complexity. 2) Balancing classification performance with accuracy, which can reduce the model’s parameters and complexity while accepting a slight decrease in classification accuracy. We can classify the networks representing these two trends as heavyweight (e.g., Vgg16) and lightweight (e.g., Resnet50, Efficientnet-B3, and MobileNetV2) networks (Hussein et al., 2023). In addition, previous studies have primarily relied on public datasets, usually characterized by enough lighting conditions, with fixed shooting angles and distances, as well as relatively simple image backgrounds. This presents a significant challenge when attempting to transition these models to natural field conditions (Ferentinos, 2018; Guo et al., 2019). Usual RGB images in complex field environments are easily affected by illumination and perspective, by which a large amount of interference makes leaf segmentation and disease identification difficult. In response to this issue, the RGB-D sensors present a practical approach. Specifically, RGB-D cameras can extract target information based on the distance of the object, making it more suitable for segmenting infected maize leaves in complex field environments (Song et al., 2022).

The above studies mainly focused on recognizing crop types based on RGB images using deep convolutional networks to improve the accuracy of recognition. However, there are few studies using RGB-D images for maize disease classification. This research focuses on the classification of three maize diseases using deep convolutional neural networks with RGB-D images. We would like to develop an approach that can be successfully integrated into portable mobile devices for timely and accurate classification of crop diseases in the field environment. To achieve this goal, we first collected the maize germplasm leaf disease data under complex field conditions using an RGB-D camera. We then segmented the captured maize leaf disease data using the RGB-D depth information to extract leaf contours and remove background interference. Later, we use four deep learning models to complete disease classification based on the pre-and post-segmentation datasets. By comparing their performances, we struck a balance between efficiency and accuracy in classification to select the best classification model. Finally, we analyzed the contribution of leaf and background in pre- and post-segmentation images on our results. This analysis aims to illuminate the stability performance of disease classification models based on pre-and post-segmentation datasets.

## 2 Materials and methods

### 2.1 Experimental site

**Figure 1:**
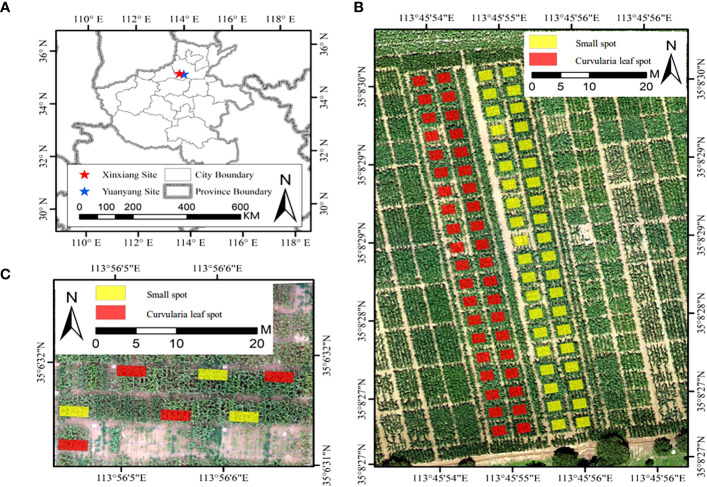
Overview of experimental sites used in this study. **(A)** The location of study sites, **(B)** the Yuanyang site, and **(C)** the Xinxiang site. Yellow and red rectangles represent the plots inoculated by the small spot and the curvularia leaf spot, respectively.

The field experiments were conducted at two sites (**Figure 1A**). Site 1 is located at the experimental base of Henan Agricultural University in Yuanyang, Henan Province (**Figure 1B**), with a mean annual temperature of 14.5°C, a mean annual precipitation of 573.5 mm, a mean annual evaporation of 1908.7 mm, a mean annual sunshine hour of 2407.7 hours, and a mean annual frost-free period of 200.5 days. Site 2 is a comprehensive experiment site, the Chinese Academy of Agricultural Sciences in Xinxiang County, Henan Province (**Figure 1C**), with a mean annual temperature of 14°C, a mean annual precipitation of 548.3 mm, a mean annual evaporation of 1748.4 mm, a mean annual sunshine hour of 2323.9 hours, and a mean annual frost-free period of 205 days. Both sites are in the Huang-Huai-Hai region, with a continental climate with abundant heat, providing a suitable environment for maize growth and disease trials.

### 2.2 Experiment design

The experiments were conducted from July to August, 2022. The Yuanyang site was planted with 20 rows for each material, and the Xinxiang site was planted with 4 rows for each material. In the Xinxiang experiment field, a total of 246 maize materials were planted, consisting of 226 maize breeding inbred lines, 8 sweet maize varieties, and 17 main cultivated varieties. The Yuanyang experiment field comprised 6 maize materials, including 5 types of maize breeding inbred lines and 1 main cultivated variety. Both test areas were inoculated with diseases: the small spot [*Bipolaris maydis* (Nishik.) Shoemaker], the curvularia leaf spot [*Curvularia lunata* (Wakker) Boedijn], and the mixed spot. Each disease was inoculated in one plot within each of the two trial areas. The disease inoculation was carried out during the maize bell stage in the evening of 2nd August, 2022. The inoculation was carried out by spraying at a spore concentration of about 100000 per ml. The amount of the inoculum in each plot was controlled at 5 ml, and the disease was evenly distributed in all plots.

### 2.3 Data acquisition

**Figure 2:**
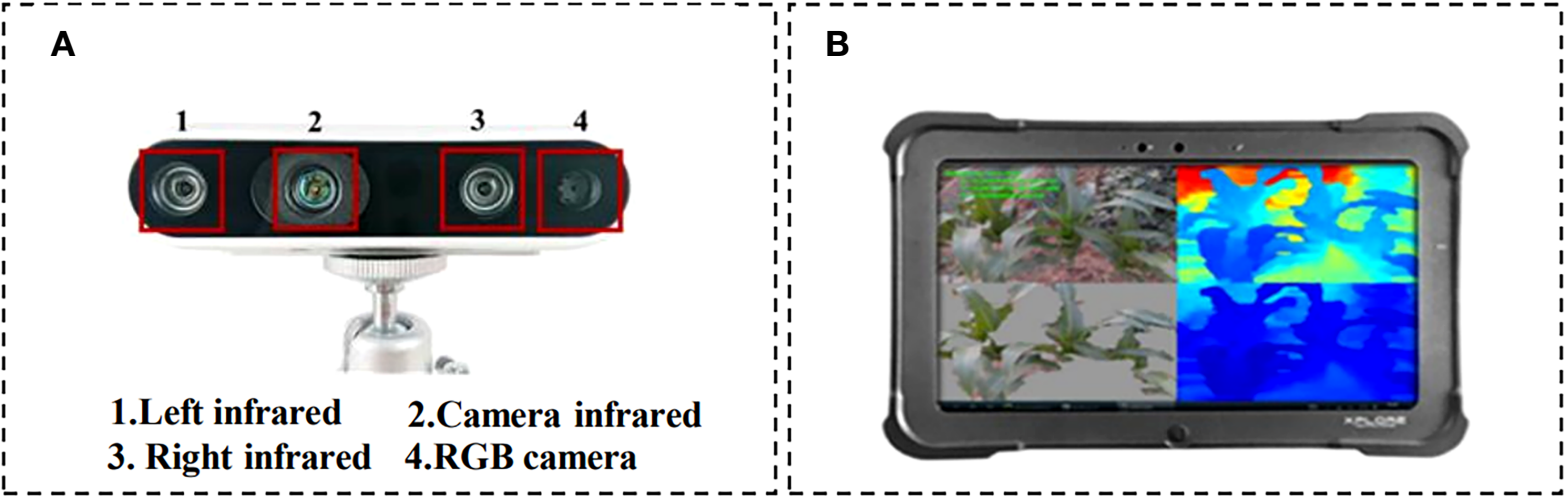
Experiment devices. **(A)** Inter RealSense D435 depth camera, and **(B)** the XPLORE tablet.

The RGB-D camera model employed for data capture is the Intel RealSense D435 (**Figure 2A**), which has a measuring range of 0.1m to 10m. The maximum resolution of images obtained through the binocular depth lens is 1280 × 720 pixels, while the maximum resolution of RGB camera is 1920 × 1080 pixels. Complementary operating software for the camera is installed on the XPLORE tablet (**Figure 2B**), with an Inter E3845 CPU and 4GB DDR3 memory running on Windows operating system. For each pathogen inoculation area, three random image collection points were selected. From each image collection point, 3–4 maize plants were chosen to capture images of infected maize leaves with handheld cameras. Our data acquisition time was mainly from 7 a.m. to 12 a.m. and 5 p.m. to 7 p.m., with a total of about 1200 images collected. During the image acquisition process, the shooting angle was not fixed to ensure the randomness of image capture and enrich the data characteristics (Khanramaki et al., 2021).

**Figure 3:**
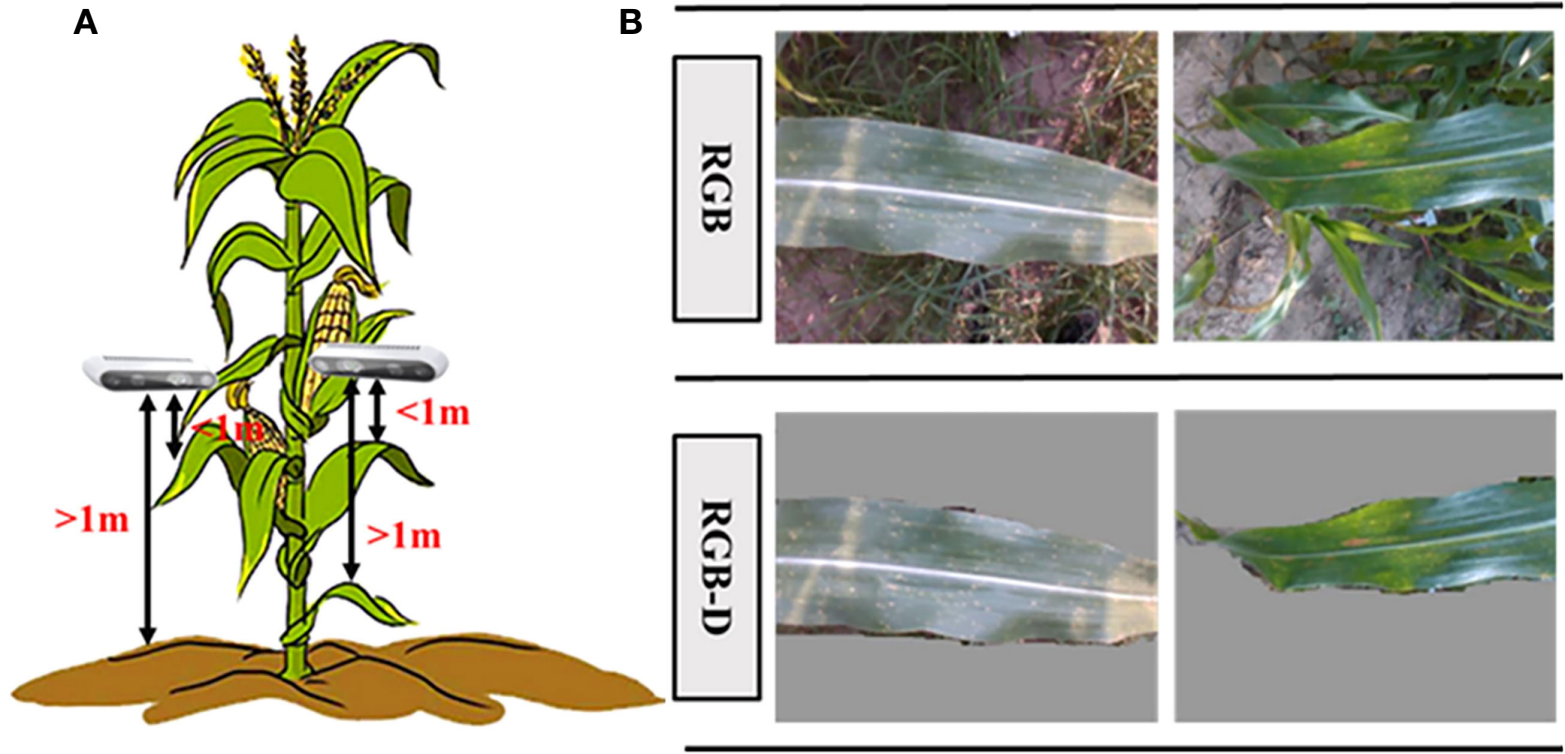
Data acquisition process in this study. **(A)** Schematic of data collection, and **(B)** the comparison of RGB and RGB-D images.

With the help of the RealSense D435 depth camara (RGB-D), RGB data of maize leaves can be obtained along with depth information. The depth information from the RGB-D image can be used to eliminate the interference from background based on distance in the depth direction, thereby reducing computational effort and improving processing speed (**Figure 3A**). In this study, background objects located one meter away from the camera were discarded (Wang et al., 2021; Lao et al., 2022). The pixels correspond to the 1-meter distance were set gray, effectively preserving the close-up maize images within one meter from the camera. **Figure 3B** presents the comparison between images captured by the RGB camera and images processed after applying the threshold using the RGB-D camera.

### 2.4 Data augmentation

**Figure 4:**
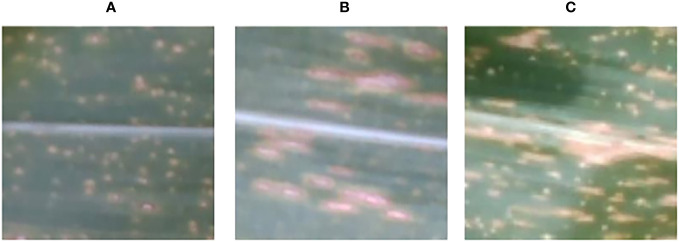
Types of maize leaf diseases, including **(A)** the curvularia leaf spot disease, **(B)** the small spot disease, and **(C)** the mixed spot disease.

**Figure 5:**
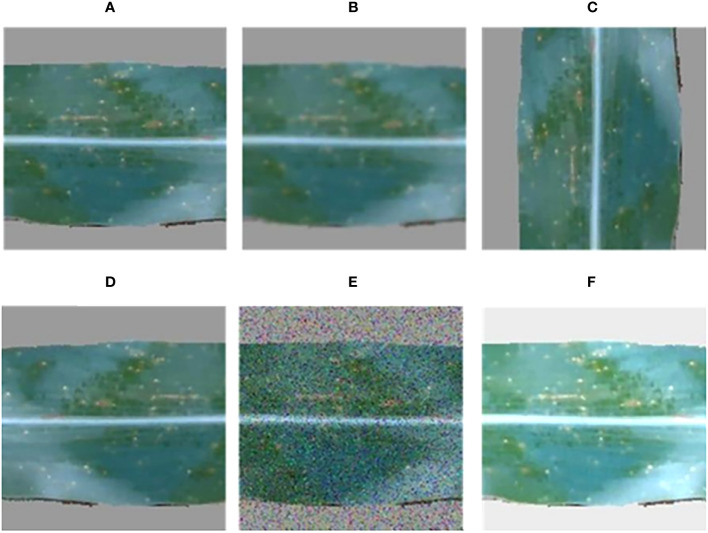
Data augmentation examples, including **(A)** original, **(B)** blurred, **(C)** rotated, **(D)** flipped, **(E)** noisy, and **(F)** brightened images.

In this study, RGB images and RGB-D segmented images were collected simultaneously using a depth camera. Two datasets, namely the pre-segmentation dataset and the post-segmentation dataset, were constructed under the same experiment conditions. Both datasets consisted of images of three maize diseases (**Figure 4**). The resolution of the images was 640 × 480 pixels, and the dataset contained approximately 1012 images in total. The pre-segmentation dataset comprised 414 images of the maize curvularia leaf spot disease, 180 images of the maize small spot disease, and 418 images of the mixed spot. The post-segmentation dataset, obtained after applying segmentation techniques, was equal in sample size. To address the issue of data imbalance and improve the generalization ability of model, data augmentation was applied. Data augmentation involves generating new data by transforming existing data and is commonly used in deep-learning models across various fields (Yu et al., 2023). In addition, we used data augmentation techniques, including original, blurred, rotated, flipped, noisy, and brightened images to expand our examples (**Figure 5**). After data augmentation, the number of samples in both the pre-segmentation and post-segmentation datasets increased to 6072 images. Then, the augmented maize leaf disease dataset was divided into the training set, test set, and validation set in a ratio of 8:1:1 ratio. Specifically, 4860, 606, and 606 samples were allocated to the training set, test set, and validation set, respectively. Both model accuracy and number of parameters were carefully considered to select the best model for classification of maize leaf diseases under field conditions.

### 2.5 Classification models

Image classification is a fundamental task in computer vision that involves categorizing images into specific semantic categories. Over the years, CNNs have emerged as highly successful models for image classification since the introduction of AlexNet in 2012 (Waheed et al., 2020). These models excel at image classification, which typically employ CNNs as backbone networks, known for their strong feature extraction capabilities (Ni et al., 2019). CNN networks are designed with various layers, including convolutional layers, pooling layers, and fully connected layers, which enable efficient extraction of significant features from the input data (Zhu et al., 2021). The convolutional layer effectively extracts local features from images, while the pooling layer down-samples and aggregates these features. Finally, the fully connected layer combines these features to form high-level features that can be used for classification or regression tasks (Paymode and Malode, 2022). In this study, we used the following models: MobilenetV2, Vgg16, Efficientnet-B3, and Resnet50.

MobileNetV2 is a lightweight convolutional neural network model suitable for environments with limited computational resources. Given the constraints of data collection and processing in field environments, MobileNetV2 was chosen to achieve high classification performance while minimizing the computational burden. Vgg16 is a classic deep convolutional neural network model that has shown excellent performance in various image classification tasks. With its deep network structure, Vgg16 can learn rich feature representations and is well-suited for classifying complex images. In the context of RGB-D data, Vgg16 can extract more comprehensive feature information from both RGB and depth images. Efficientnet-B3 is a series of convolutional neural network models based on automated model scaling methods. Efficientnet-B3 strikes a good balance between model size and computational resource consumption. It achieves high performance while having fewer parameters, making it suitable for corn leaf disease classification tasks with limited computational resources. Resnet50 is a deep convolutional neural network model with residual connections. It effectively addresses the issue of gradient vanishing that can arise from deep networks, resulting in better training performance. Resnet50 is suitable for image classification tasks in complex environments and can capture rich feature representations from multiple perspectives when handling RGB-D data.

These selected models have demonstrated strong performance in image classification tasks and are well-suited for classifying corn leaf diseases in complex field environments based on RGB-D data. The choice of these specific models aims to balance classification performance while considering computational resources and model size limitations. By comparing the performance of these models on training and validation datasets, researchers can select the best model for accomplishing the corn leaf disease classification task.

#### 2.5.1 MobilenetV2

MobilenetV2 (Indraswari et al., 2022) is a lightweight neural network as an improvement over MobileNetV1 introduced by the Google team in 2018. It retains the deep separable convolution concept of MobileNetV1 while introducing linear bottleneck and inverse residual structures. MobilenetV2 utilizes a deeply separable convolutional network design, combining standard convolutional layers with deeply separable convolutional layers. This design significantly reduces the number of network parameters and computational effort while maintaining accuracy. Additionally, MobilenetV2 introduces a linear bottleneck structure in the network architecture, which reduces the number of parameters in the depth dimension. To further enhance performance and computational efficiency, MobilenetV2 incorporates techniques such as residual blocks and asynchronous data batching. The residual blocks accelerate model convergence, minimize the risk of overfitting, and optimize the speed of forward inference. Asynchronous data batching overlaps computation and communication execution to improve parallelism and data throughput in the model.

#### 2.5.2 Vgg16

Vgg16 (Kristiani et al., 2020; Raghu et al., 2020; Rangarajan and Purushothaman, 2020) is a well-known convolutional neural network model in deep learning. It was developed by researchers at the University of Oxford and achieved the second-place ranking in the 2014 ImageNet Large Scale Image Recognition Competition (ILSVRC). The name “Vgg16” comes from the structure of the network, which consists of 16 convolutional layers and 3 fully connected layers. In the convolutional layers, Vgg16 uses 3 × 3 convolutional kernels with a stride of 1 and padding set to “same”. The ReLU activation function is applied after each convolutional operation. The output of the convolutional layers is then down sampled using pooling layers to reduce the size of the feature maps. At the top of the network, the feature maps are mapped to the 1000 categories of the ImageNet dataset using a fully connected layer. One advantage of Vgg16 lies in its simple and easy-to-understand network structure, which makes it straightforward to implement. It also performs well on various visual tasks. However, there are some disadvantages to consider. Vgg16 has many parameters, which requires higher computational resources and training time. Additionally, the model is prone to overfitting issues, where it may struggle to generalize well to unseen data. Overall, Vgg16 is a powerful model that has made significant contributions to image recognition tasks, but it should be used with consideration for its computational requirements and potential overfitting challenges.

#### 2.5.3 Efficientnet-B3

Efficientnet-B3 (Zhang et al., 2020; Atila et al., 2021) is an efficient convolutional neural network architecture proposed by the Google team in 2019. It combines innovative neural network design principles with automated model scaling techniques to improve model accuracy while reducing the number of parameters and computational requirements. Efficientnet-B3 is a member of the Efficientnet family, specifically a larger variant. It has demonstrated excellent performance on the ImageNet dataset. The network structure of Efficientnet-B3 consists of repetitive modules that include deep separable convolution, dilated convolution, and normal convolution operations. One of the key features of Efficientnet is its automated model scaling approach. This approach scales the network architecture based on factors such as depth, width, and resolution. Composite coefficients are used to balance these three factors during the scaling process. By leveraging this method, Efficientnet achieves higher accuracy while keeping the model’s parameters and computational complexity relatively small. Efficientnet represents a significant advancement in building efficient and accurate convolutional neural network architectures. Its innovative design and scaling technique make it a powerful tool for various computer vision tasks while minimizing the resource requirements.

#### 2.5.4 Resnet50

Resnet50 (Tan et al., 2018; Yu et al., 2023) is a deep residual network model that was proposed by the Microsoft Asia Research Institute. It is considered one of the classical models in the Resnet family and has gained significant popularity as one of the most commonly used convolutional neural network (CNN) models. The main concept behind Resnet50 is residual learning, which introduces shortcut connections in the CNN architecture to learn a residual mapping. This approach addresses the problem of vanishing gradients that can occur during the training of deep networks. By using residual connections, Resnet50 enables effective training of deep networks and improves the accuracy of the model. The network structure of Resnet50 is relatively deep, consisting of 50 convolutional layers. It includes 16 convolutional modules. The first module performs initial feature extraction using 7x7 convolutional layers and pooling layers. The subsequent modules are composed of residual blocks, each consisting of two convolutional layers and a residual connection. In the final fully-connected layer, Resnet50 maps the feature maps generated by the convolutional layers to the 1000 categories of the ImageNet dataset. The Resnet50 achieves better performance by increasing both the depth and computational complexity of the model. It is widely used in various computer vision tasks, such as image classification, object detection, and semantic segmentation. Resnet50 has demonstrated excellent results on the ImageNet dataset and has become one of the most popular deep-learning models in use today.

### 2.6 Class activation mapping

We analyzed the contribution of leaves and background in both pre-segmented and post-segmented images using Class Activation Mapping (CAM) (Feng et al., 2023). Class Activation Mapping (CAM) represents a technique within deep learning, particularly in the realm of Convolutional Neural Networks (CNNs). CAM enables visual interpretability of CNN decision-making by spatially highlighting the influential regions in an input image that contribute most to a specific class prediction.

The implementation process of Class Activation Mapping (CAM) is as follows: 1) Train the CNN: Begin by training the Convolutional Neural Network (CNN) conventionally using a dataset tailored to a specific classification task. 2) Remove the FC layers: After training, strip away the fully connected (FC) layers that succeed the final convolutional layer. This reveals the spatial dimensions of the concluding feature maps, which will be crucial for visual interpretation. 3) Compute the CAM: To generate the Class Activation Map for a class, c, one must calculate a weighted summation of the activations extracted from the feature maps of the last convolutional layer. The weights determine the significance of these feature maps with respect to class c. The formula for computing CAM is:


(1) $$\text{CA}{\text{M}}^{\text{c}}\left(\text{x},\text{y}\right)=\sum_{\text{k}}{\text{w}}_{\text{k}}^{\text{c}}{\text{F}}_{\text{k}}\left(\text{x},\text{y}\right)$$

where $CAM^{c}\left(x,y\right)$ represents the class activation map’s value for class c at the spatial location (x, y), $w_{k}^{c}$ symbolizes the weightage of the $k^{th}$ feature map for class c, $F_{k}$ (x, y) is the activation at location (x, y) for the $k^{th}$ feature map. Using CAM, researchers can visually understand which regions in an image have been crucial for the CNN in making its decision for a class. This method provides an intuitive way to inspect and interpret the workings of a trained CNN model.

### 2.7 Evaluation metrics

The confusion matrix, also known as the error matrix, is a standard evaluation metric indicating the classification accuracy and is represented in the form of a matrix with n rows and n columns, as shown in **Table 1** (Yu et al., 2022). The “Accuracy”, “Precision”, “Recall”, and “F1-Score” were selected to evaluate the model performance comprehensively (Wu et al., 2020).

**Table 1:** Example of a confusion matrix.

Confusion matrix		Ture label	
		Positive	Negative
Predicted label	Positive	TP	FP
	Negative	FN	TN

TP, True Positive; TN, True Negative; FP, False Positive; and FN, False Negative.

Accuracy is the ratio of the number of samples correctly classified by the model to the total number of samples. The higher the precision, the higher the classification accuracy of the model. The precision rate is the ratio of the number of samples that the model correctly predicts as positive cases to the number of all samples that are predicted as positive cases. The higher the precision rate, the more accurate the model is in predicting positive cases. Recall is the ratio of the number of samples that the model correctly predicts as positive cases to the number of all samples that are positive cases. The higher the recall, the better the ability of the model to correctly identify the cases that are positive. The F1-score combines the metrics of accuracy and recall and is a comprehensive evaluation metric. the higher the F1-score, the better the classification accuracy and ability to identify actual positive examples of the model. The expressions are as follows:


(2) $$Accuracy=\frac{\text{TP}+\text{TN}}{\text{TP}+\text{TN}+\text{FP}+\text{FN}}$$

(3) $$Precision=\frac{\text{TP}}{\text{TP}+\text{FP}}$$

(4) $$Recall=\frac{\text{TP}}{\text{TP}+\text{FN}}$$

(5) $$\text{F}1-\text{Score}=2*\frac{\text{Precision}*\text{Recall}}{\text{Precision}+\text{Recall}}$$

### 2.8 Model training

The computer equipment used in this study consisted of the Intel(R) Xeon(R) Gold 5218 CPU with 2.3 GHz, the NVIDIA GeForce RTX 3090 GPU with 24 GB of video memory, and the 128 GB of RAM. The experiment was conducted using PyTorch 1.13.1 framework, CUDA version 12.0, and cuDNN version 7.6.5. The accuracy and loss values were saved after each epoch during the training process, which allows for visual inspection of the convergence for model. It is worth noting that conclusions only drawn from the converged state hold reference value. The experimental parameters are set as illustrated in **Table 2** (Leo, 2023). The cross-entropy loss function, as described by Yang et al. (2023), is a commonly used loss function in classification tasks. It quantifies the disparity between the predicted outcomes of a model and the true labels. The cross-entropy loss function evaluates the dissimilarity between the probability distribution predicted by the model and the actual distribution. As a result, it serves as a crucial metric for assessing the classification performance of the model. During the training process, the objective is to optimize the model parameters by minimizing the loss function. This optimization aims to enhance the prediction accuracy of the model. By utilizing the cross-entropy loss function as the optimization objective, the model can make more accurate predictions and obtain optimal model parameters.

**Table 2:** Experimental parameter settings.

Parameter name	Parameter value
Learning rate	0.0001
Optimizer	SGD
Momentum parameter	0.90
Batch size	16
Epoch	500

For all four models, the initial learning rate is set to 0.0001, the momentum parameter is set to 0.9, and stochastic gradient descent (SGD) is chosen as the optimizer. The learning rate determines how quickly the weight values are updated. If the learning rate is set too high, it may result in overshooting the optimal value, while an excessively low learning rate can slow down the convergence rate. Manually adjusting the learning rate requires constant fine-tuning. Hence, the introduction of momentum parameters assists SGD in achieving faster learning rates. Additionally, to further optimize the learning rate at regular intervals, the StepLR method is applied with defined step size and gamma parameters of 7 and 0.1, respectively. This helps to modify the learning rate at fixed intervals (Paszke et al., 2017).

## 3 Results

### 3.1 Model performance for maize leaf disease classification

Considering the future deployment of the model on mobile devices, it is important to evaluate the limited computing power and other constraints inherent in such devices. Therefore, this study incorporated additional evaluation metrics such as the memory footprint of the model, the time required to predict a single instance on a mobile device, and the average prediction time per instance. For the four deep learning models, i.e., MobilenetV2, Vgg16, Efficientnet-B3, and Resnet50, we evaluated various performance metrics, including “Accuracy”, “Precision”, “Recall”, and “F1-Score”. We also analyzed the mean prediction time for each deep learning model before and after segmentation. These evaluation metrics allow for a comprehensive assessment of the model’s performance in a mobile device environment.

For the dataset with the pre-segmentation process (**Table 3**), the Resnet50 model exhibited the best classification performance with an F1-score of 98.53%, an Accuracy of 98.16%, a Precision of 98.53%, and a Recall of 98.53%. However, it should be noted that this model had a larger model size of 45.0 MB. On the one hand, although the classification results were slightly lower than those of Resnet50, the size of the MobilenetV2 model was greatly reduced to 4.44 MB, which was about one-tenth of that of Resnet50. On the other hand, it required less computational power and ran smoothly on mobile devices. Therefore, in terms of overall classification performance, the MobilenetV2 model (F1-score = 97.82%, Accuracy = 97.53%, Precision = 97.82%, Recall = 97.82%) was more suitable for deployment on mobile devices.

**Table 3:** Comparison of pre-segmentation model results.

Models	F1-Score	Accuracy	Precision	Recall	Run time	Model size
MobilenetV2	97.82%	97.53%	97.82%	97.82%	1.24s	4.44 MB
Vgg16	96.92%	96.87%	97.83%	96.57%	3.33s	256.00 MB
Efficientnet-B3	97.78%	97.69%	97.47%	98.14%	1.84s	20.90 MB
Resnet50	98.53%	98.16%	98.53%	98.53%	2.10s	45.00 MB

For the dataset after segmentation (**Table 4**), the classification accuracies of the Resnet50 model (F1-score = 98.22%, accuracy = 98.02%) and the MobilenetV2 model (F1-score = 98.22%, accuracy = 98.02%) were essentially the same, with only a 0.02% difference in accuracy. Compared with the disease classification models constructed based on the pre-segmented dataset, the Resnet50 and Vgg16 models constructed based on the post-segmented dataset showed a slight decrease in accuracy, while the Efficientnet-B3 and MobilenetV2 models showed a slight increase in accuracy.

**Table 4:** Comparison of post-segmentation model results.

Models	F1-Score	Accuracy	Precision	Recall	Run time	Model size
MobilenetV2	98.22%	98.02%	98.22%	98.22%	1.21s	4.44 MB
Vgg16	96.17%	96.37%	96.37%	95.99%	2.75s	256.00 MB
Efficientnet-B3	98.09%	97.86%	97.92%	98.26%	1.65s	20.90 MB
Resnet50	98.22%	98.02%	98.24%	98.22%	1.71s	45.00 MB

### 3.2 Confusion matrices before and after segmentation

**Figure 6:**
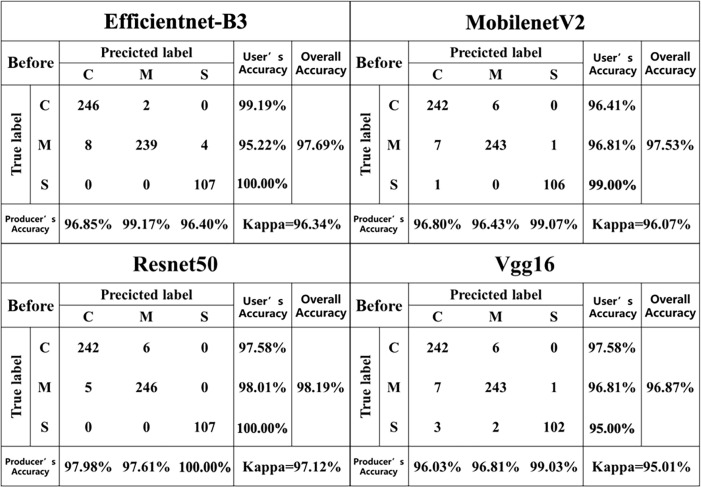
Confusion matrices before segmentation for each model.

**Figure 7:**
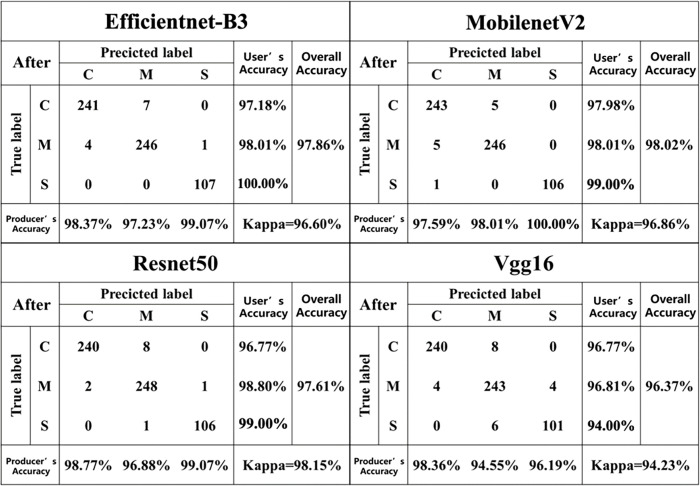
Confusion matrices after segmentation for each model.

**Figures 6**, **7** describes the confusion matrices for the pre- and post-classification test sets of the MobilenetV2, Vgg16, Efficientnet-B3 and Resnet50 deep learning models for the three diseases. For the pre-segmentation dataset, the Resnet50 model performed better than the other models in classifying the mixed disease speckle dataset with respect to the pre-segmented dataset, while MobilenetV2 ranked second and Vgg16 performed poorly. Notably, the Vgg16 results performed inconsistently in identifying maize small spot disease, with an accuracy of only 95.00%. Among the misclassifications, 2.80% of the small spot test samples were misclassified as the curvularia leaf spot, and 1.87% of the small spot and 2.48% of the curvularia leaf spot test samples were misclassified as the mixed spot. In addition, 2.79% of the mixed spot test samples were misclassified as the curvularia leaf spot. The reason for this discrepancy may be the similarity in early characteristics of the small spot and the curvularia leaf spot, thus confusing the classifiers. In addition, the curvularia leaf spot had the highest classification accuracy, with the classifier correctly identifying 97.58% of the mixed spot test samples, demonstrating excellent performance. In addition, the classifier’s classification accuracy for the mixed spots has been consistent, correctly classifying 96.87% of the test samples.

### 3.3 Prediction time before and after segmentation

**Figure 8:**
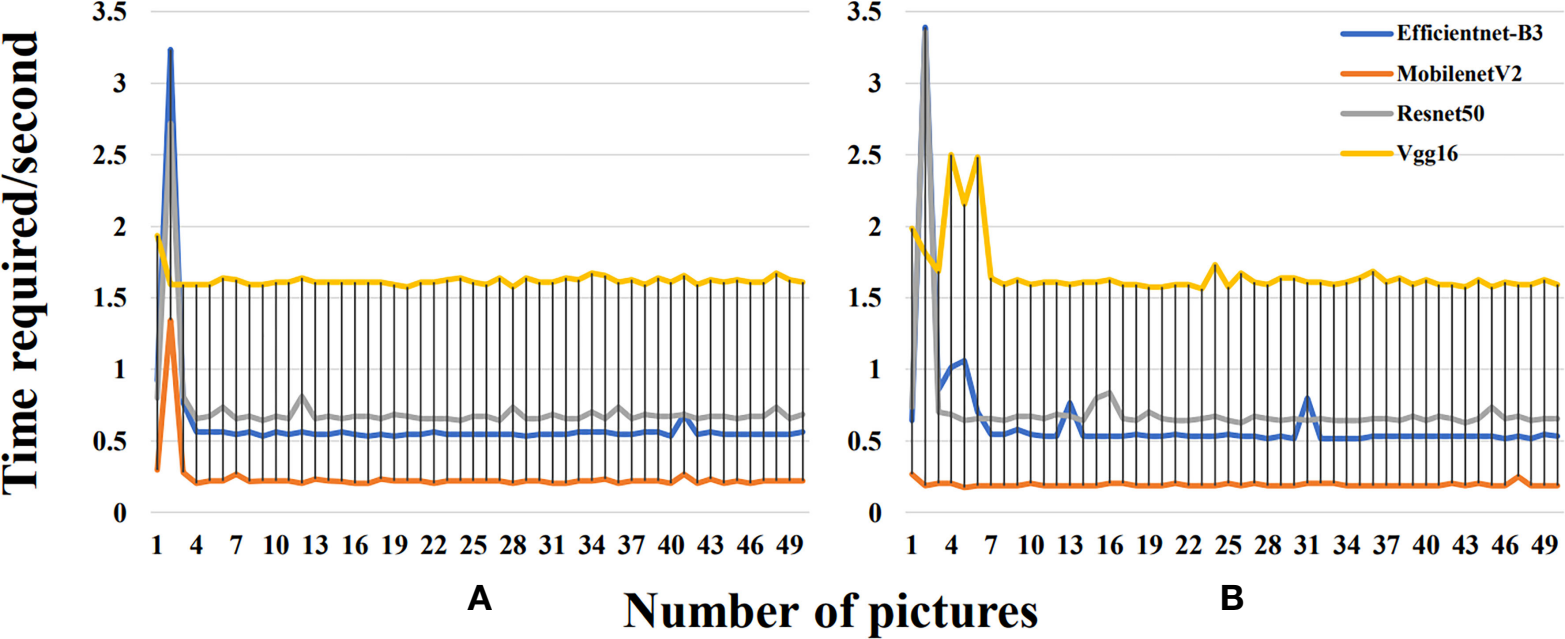
**(A)** Time required for a single prediction before segmentation for each model. **(B)** Time required for a single prediction after segmentation for each model.

This study further compared the temporal curves of the single prediction before and after segmentation of 50 images with the same disease in the validation set using four deep learning models (MobilenetV2, Vgg16, Efficientnet-B3, and Resnet50) (**Figure 8**). During the prediction process of these deep learning models, we observed a clear pattern of temporal changes. Initially, when the deep learning models started making predictions, they took more time due to the high computational complexity of the models, the time-consuming training process, and the initial lack of model adaptation to the data. However, after enough training, these models gradually acquired more accurate feature representations and more efficient parameter combinations, which significantly reduced the prediction time. The time required after segmentation was slightly reduced compared to that before segmentation. The image segmentation operation can simplify the complexity of the input image, which is conducive to better recognition and understanding of image features by the neural network. By removing some unnecessary information, the efficiency of the model during training and inference can be improved, thus decreasing training time and resource consumption. By segmenting the background, the neural network only focused on the region containing the object without having to deal with the whole region. The reduction in computation can speed up the model inference. Especially, in the real world, where most of the objects are concentrated in the center of the image, it is also possible to reduce the processing area and thus obtain more accurate results. Overall, the Resnet50 model achieved excellent performance in classifying the mixed pot dataset in the test samples of the segmented dataset, followed by MobilenetV2. However, Vgg16 performed poorly in this regard. It is worth noting that the accuracy of the Vgg16 and Resnet50 models decreased after segmentation, while the accuracy of MobilenetV2 and Efficientnet-B3 increased. Taken together, the MobilenetV2 model performed better than the other three models in terms of the size and the single image prediction time.

## 4 Discussion

**Figure 9:**
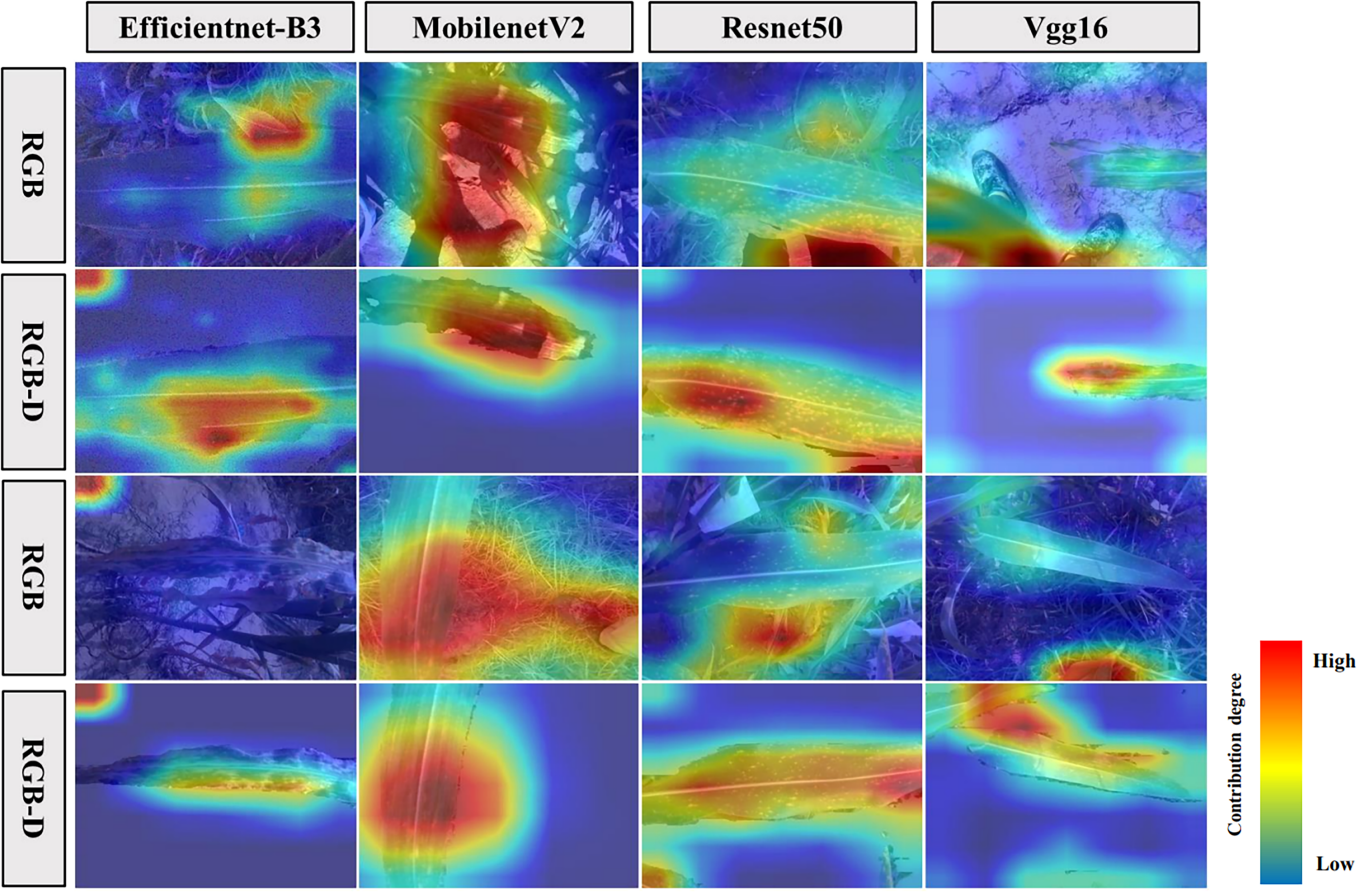
Comparison of feature extraction visualization on images before and after segmentation for each model.

To further discuss the factors contributing to the variation in classification accuracy between models trained on pre-segmentation and post-segmentation datasets, this study also explored the causes through feature visualization techniques (**Figure 9**). We found that the pre-segmentation images not only contained leaf and disease spot features but also included noise features such as soil, weeds, and stems. Compared to previous studies, it was observed that besides the leaf features contributing to the background, the noise features played an even more significant role (Ferentinos, 2018). In addition, the variations in soil background and other factors were found among different plots, resulting in a degradation of model performance when applied to different plots or different years (Guo et al., 2019). The leaf region contributed more to the segmented images, that is, the extracted classification features were mainly from the leaf. Consequently, this reduced the impact on performance when the model was applied to different plots or different years (Song et al., 2022).

The Vgg16 (Raghu et al., 2020) and Resnet50 (Tan et al., 2018; Yu et al., 2023) models are deep neural networks characterized by many parameters and complex network structures. When dealing with pre-segmentation images, these models tend to extract features not only from the disease but also from the complex backgrounds. This may lead to the models considering the complex backgrounds as additional information for disease recognition, resulting in improved accuracy for disease recognition in the pre-segmentation images compared to post-segmentation images (Chen et al., 2019). In contrast, MobilenetV2 (Indraswari et al., 2022) and Efficientnet-B3 (Zhang et al., 2020; Atila et al., 2021) are lightweight networks with fewer parameters and simpler network structures. When extracting disease features from pre-segmentation images, these models may be limited by the complex backgrounds without considering relevant disease features. However, in the post-segmentation images, these models can focus on extracting disease-specific features after removing the influence of the complex background (Wang et al., 2021; Lao et al., 2022). As a result, these models exhibited lower accuracy for disease recognition in the pre-segmentation images compared to the post-segmentation images. For contexts requiring swift outcomes, such as on-field applications or embedded systems, lightweight models like MobilenetV2 and Efficientnet-B3 might be more apt. Specifically, the MobilenetV2 model, when processing post-segmented images - especially after the removal of intricate backgrounds - focuses on disease-specific features. This holds invaluable real-world merit, as in many instances, interest might be concentrated solely on the disease, discounting its surroundings.

Deep learning models typically employ feature mapping to characterize image information, and increasing the network depth generally improves accuracy (Liu et al., 2018). However, this also leads to a higher number of parameters and computational complexity. In identifying the small spot and curvularia leaf spot diseases, these spots tend to exhibit similar pixel patterns, requiring higher resolution representations than those extracted from CNN feature maps alone (Waheed et al., 2020). Although increasing the depth of the network results in more feature maps, these feature maps become narrower as the network grows and may result in ambiguous information about the spots. Considering that there is no direct computational relationship between the spots, simply increasing the number of network layers would not necessarily improve the accuracy, as evidenced by the low classification accuracy of Vgg16 (Kristiani et al., 2020). In contrast, the Resnet50 model utilizes residual connections facilitates learning residual mappings and addresses the vanishing gradient problem, allowing for effective feature extraction This design choice helps the network effectively learn input features, which is the reason why Resnet50 achieved the highest accuracy in this study (He et al., 2016).

While Resnet50 exhibited superior accuracy, it falls short in terms of operational speed compared to MobilenetV2 and Efficientnet-B3. Both MobilenetV2 and Efficientnet-B3 are lightweight networks, with the latter being deeper and more complex than the former. To maintain efficiency while preserving a certain level of accuracy, Efficientnet-B3 employs a composite scaling method that uniformly scales the width, depth, and resolution of the network. However, the increased complexity in the design of Efficientnet-B3 also results in higher computational requirements, potentially leading to longer execution times compared to MobilenetV2 (Joohyung et al., 2022).

To validate the classification performance of the method proposed in this study, we compared the optimal result of this paper, MobilenetV2, with SKPSNet-50 (Zeng et al., 2022), the improved yolov5n (Ma et al., 2023), and the improved ShuffleNet V2 (Fei et al., 2023) for crop disease classification or crop classification. The SKPSNet-50 proposed model is more effective in recognizing corn leaf diseases in natural scene images, which has fewer parameters and computation compared to the heavy-duty model, but still has a higher number of parameters compared to some lightweight networks, and its average recognition accuracy is 92.9%, which is about 50% higher than the SKNet-6 model. By adding CA (Coordinate Attention) attention module to the Yolov5n lightweight model, it improves 2.8 percentage points since the original model, and the average recognition accuracy can be up to 98.20%, which effectively improves the recognition accuracy of the not easily recognizable maize grey spot and maize big spot diseases, and the method is characterized by higher accuracy and smaller size. In the classification study of fresh cut flowers by the improved ShuffleNet V2, the classification accuracy based on the traditional color image dataset reaches 98.89%, and the classification accuracy based on the deep data dataset is as high as 99.92%, and the classification speed of each flower reaches 0.020 s. Our MobilenetV2 result has a classification accuracy of up to 98.22% for segmented images, and the model size is only 4.44 MB with a classification speed of 1.21 s. This method is robust to classify corn disease images collected in natural environments and can provide high accuracy with limited computational resources. However, its recognition efficiency is still low since low computational loads. We believe that there is potential for further improving its accuracy and efficiency in the future.

Therefore, it is important to consider the trade-off between computational efficiency and accuracy when selecting a suitable model for specific tasks or constraints. MobilenetV2 (Indraswari et al., 2022) strikes a successful balance between accuracy and efficiency through its lightweight design, optimized network structure, and utilization of scaling strategies. It achieves high classification accuracy while imposing a low computational load, making it particularly well-suited for computing resource-constrained environments (Paymode and Malode, 2022). This characteristic renders MobilenetV2 an ideal choice for scenarios that involve limited resources, such as mobile devices and embedded systems.

With the evolving panorama of mobile technology and IoT devices, lightweight models demanding fewer resources, like MobilenetV2, can find extensive applications in these devices. Farmers employing a straightforward mobile app, capturing pictures of maize leaves with their smartphone cameras, followed by the app swiftly identifying and furnishing information about the disease, along with recommended treatment measures. Furthermore, as UAV technology garners popularity in agriculture, our algorithms can be integrated into these UAVs for real-time monitoring of vast agricultural expanses, ensuring quicker identification and treatment of diseases. Fundamental constraint in our research was the undersupply of samples characteristic of healthy maize leaves. This shortfall has indeed affected the comprehensive spectrum of our dataset. We emphasize the need for future investigations to channel efforts towards collating and incorporating a richer array of genuine healthy leaf samples. Such a holistic approach would inevitably enhance the trustworthiness and broader applicability of the classification frameworks. Our current emphasis on maize-centric diseases, though insightful, has given us a deeper appreciation for the wider scope and adaptability of our techniques across different crops. We are eager to gauge the performance of our deep learning paradigms in discerning diseases across a variety of crops. This exploration serves a dual purpose: it underscores the adaptability and resilience of our models and magnifies their relevance in an extended agricultural panorama.

## 5 Conclusions

This study presented a novel method for maize leaf disease classification using the RGB-D post-segmentation image data. To enhance robustness and generalization, a data preprocessing method including accurate edge detection, cropping and data enhancement is designed. We trained four maize disease classification models (i.e., Resnet50, MobilenetV2, Vgg16, and Efficientnet-B3) with the deep learning approach to identify three main maize diseases, including the curvularia leaf spot, the small spot, and the mixed spot diseases. The experimental results show that the classification accuracy of the deep image dataset reaches 97.82% with limited sample size and less hardware resources, and the classification speed of each flower can be up to 1.21 s. Compared with other traditional lightweight classical networks, the proposed method shows strong competitiveness and excellent classification performance in terms of the model size, the number of parameters, the recognition accuracy, and the image detection speed. The MobilenetV2 model performed better than the other three models in terms of the size and the single image prediction time. The higher classification accuracy and a better stability can help develop better tools for agricultural disease detection. Considering the limited number of disease species used in this study, there may be other factors affecting the accuracy of the model in practical applications, it is necessary to enrich the dataset by collecting more healthy and diseased leaves in the future to enhance the robustness of the results. To further improve the efficiency of disease identification and classification, more research targeting large-scale field tasks is still needed. Overall, our findings offer the possibility of developing relevant portable devices to improve the selection efficiency of the next generation of disease-resistant crop resources. In the future, we will conduct further research on the classification system of crop diseases, including not only corn, but also soybeans, wheat, potatoes, etc. to expand the application areas and improve the practical application value of the classification system.

## Data availability statement

All the raw data and code can be available from the corresponding authors upon reasonable request. Requests to access these datasets should be directed to XJ, jinxiuliang@caas.cn.

## Author contributions

FN: Writing – original draft, Writing – review & editing, Data curation, Investigation, Software, Validation, Visualization. YS: Writing – original draft, Writing – review & editing, Conceptualization, Formal Analysis, Methodology, Supervision. XY: Data curation, Validation, Visualization, Writing – review & editing. CN: Data curation, Validation, Visualization, Writing – review & editing. YL: Data curation, Validation, Visualization, Writing – review & editing. YB: Investigation, Resources, Writing – review & editing. DZ: Investigation, Resources, Writing – review & editing. CW: Methodology, Project administration, Resources, Writing – review & editing. DY: Methodology, Project administration, Resources, Writing – review & editing. WY: Funding acquisition, Project administration, Resources, Supervision, Writing – review & editing. XJ: Funding acquisition, Project administration, Resources, Supervision, Writing – review & editing.
